# Anastomosis selection in liver transplantation for recipients with unusable recipient hepatic arteries: a bayesian network meta-analysis

**DOI:** 10.1186/s12893-024-02385-4

**Published:** 2024-03-23

**Authors:** Warsinggih, Citra Aryanti, Julianus Aboyaman Uwuratuw, Erwin Syarifuddin, Ronald Erasio Lusikooy, Muhammad Faruk

**Affiliations:** 1 Division of Digestive, Department of Surgery, Faculty of Medicine, Hasanuddin University, Jalan Perintis Kemerdekaan KM 11, Makassar, 90245 South Sulawesi Indonesia; 2 Department of Surgery, Faculty of Medicine, Hasanuddin University, Makassar, Indonesia

**Keywords:** Hepatic artery, Liver transplantation, Splenic artery

## Abstract

**Background:**

The anastomosis of donor and recipient hepatic arteries is standard in liver transplantations. For transplant recipients with unusable hepatic arteries, appropriate artery selection should be conducted using evidence-based considerations; therefore, this network meta-analysis (NMA) aimed to analyze the most suitable alternative recipient artery for anastomosis during liver transplantations.

**Methods:**

Comprehensive searches of the Scopus, Cochrane Library, and MEDLINE databases were conducted to analyze observational studies containing non-standard anastomoses in liver transplantations that used the splenic artery, aorta, celiac, or branches of the gastric artery. The outcome parameters included intraoperative components, complications, and survival data. This NMA used the BUGSnet package in R studio and the results were presented in a Forest plot, league table, and SUCRA plot.

**Results:**

Among the 13 studies included in this NMA, 5 arteries were used for the anastomoses. The splenic artery anastomosis showed a high risk of thrombosis and a low risk of stenosis (OR 1.12, 95% CI 0.13–3.14) and biliary tract abnormalities (OR 0.79, 95% CI 0.36–1.55). In addition, the graft survival (OR 1.08; 95% CI 0.96–1.23) and overall survival (1-year survival OR 1.09, 95% CI 0.94–1.26; 5-year survival OR 1.95% CI 0.83–1.22) showed favorable results using this artery. Constraints to the use of the splenic artery were longer operation and cold ischemic times. However, the duration of hospital stay (MD 1.36, 95% CI -7.47 to 10.8) was shorter than that when the other arteries were used, and the need for blood transfusions was minimal (MD -1.74, 95% CI -10.2 to 6.7).

**Conclusion:**

In recipients with unusable hepatic arteries, the splenic artery of the patient should be the first consideration for anastomosis selection in liver transplantations.

## Background

Liver transplantation was initiated in 1958 by Moore [1] and has become a surgical procedure that offers hope and renewed life to patients suffering from end-stage liver disease or acute liver failure [2]. Worldwide, the number of liver transplants has been steadily increasing by 6.5% annually, with 34 694 procedures performed in 2021, of which 23% involved living donors [3]. In Asia, liver transplantations have been largely performed in South Korea [4], where 1543 surgeries were conducted in 2019, with a 70% survival rate of ten-years comparable to other countries survival rate [5].

The hepatic artery is a vital conduit for ensuring proper blood supply to the transplanted liver [6]. However, the hepatic artery of the transplant recipient is not always a viable option, owing to intimal dissection, complete thrombosis, inadequate flow or small size, or difficult variations. Therefore, alternative conduits can be substituted, such as the splenic artery, aorta, celiac, or branches of the gastric artery [7].

Although several studies have compared the outcomes using alternative conduits, no specific artery has been chosen as the standard. By exploring the outcomes and complications, this network meta-analysis aimed to determine the intraoperative measures, complications, and survival parameters among splenic, celiac, aortic, and gastric arteries as alternative recipient anastomoses in liver transplantations.

## Materials and methods

### Study design

The network meta-analysis (NMA) was conducted according to the Preferred Reporting Items for Systematic Review and Meta-Analysis Protocols (PRISMA-P) and additional NMA extension guidance [8, 9]. The purpose of the literature search was to address the following research question formulated using the PICO framework: Population (liver transplant recipient with unusable hepatic artery), Interventions (Splenic, celiac, aortic, and gastric arteries), Comparison (Hepatic artery), and Outcome (Intraoperative measures: operation time, cold ischemic time, blood transfusion requirement, length of hospital stay; Complications: thrombosis, stenosis, bile duct complications; and Survival: patient and graft survival). This study was registered in PROSPERO with registration number CRD42023432987.

### Literature search

Comprehensive article and related citation searches were performed using PubMed with specific keywords. The articles were updated to May 30, 2023. Although quasi-randomized clinical trials (RCTs), RCTs, and observational studies were eligible, no RCTs were identified. No restrictions were placed on the publishing year, date, or status of the work. The inclusion criteria were: (1) studies comparing anastomoses using the donor hepatic artery to those with alternative conduits (splenic, gastric, celiac, or aortic artery) during liver transplantations, (2) studies that met the GRADE Working Group’s benchmark for a good study, and (3) randomized controlled trials or prospective studies. The exclusion criteria were studies that were (1) lacking exact survival data, (2), conducted with mice, or (3) not in the English language.

### Study selection

Each title and abstract was independently read. After completion of the search, duplicates were eliminated and the papers were assessed. Abstracts that appeared in several searches as possible matches for inclusion were read. The full-text articles were examined to ascertain eligibility when all possible eligible studies were retrieved. The investigators discussed and resolved any inconsistencies and authors were notified if any information appeared to be missing.

### Data extraction

The following data were extracted using a Microsoft Excel standardized electronic data form: author name, country, study year, and artery type used, and expected outcomes were tabulated. Two independent reviewers extracted the data from the included studies to minimize bias, while a third verified the data to prevent repeat inclusion.

### Risk of bias assessment

The Cochrane risk of bias tool was used to evaluate the studies for bias, taking into account factors such as selective reporting, insufficient outcome data, participant blinding, allocation concealment blinding, random sequence creation, and other potential sources of bias. The parameters were then categorized as having a “Low risk”, “High risk”, or “Unclear risk” of bias [10].

### GRADE assessment

The GRADE Working Group method grades the quality of treatment effect estimates from network meta-analyses and was used to interpret the evidence. This method involves evaluating the quality of each NMA effect estimate, direct and indirect treatment estimates for each comparison of the evidence network, rating the NMA estimate for each evidence network comparison, and assessing the direct and indirect treatment estimates of each comparison.

### Data synthesis

A narrative summary of the chosen studies was presented. Following data collection, the various outcomes were tabulated and classifications were formed based on the study characteristics, demographics, and type of therapy used.

### Network meta-analysis

The network meta-analysis was conducted using a Bayesian framework and the BUGSnet package of R software version 1.1.0 [11]. The NMA model was performed using a Bayesian approach with the Markov Chain Monte Carlo (MCMC) simulation. The Bayesian framework had 10 000 iterations, 1000 burn-ins, and 1000 adaptations. Deviance information criteria (DIC) were used to assess the goodness-of-fit and selection of the model. A close match between the two models was deemed to be an adequate fit, and the adequacy of the fit was determined by comparing the residual deviation of the models. We conducted a SUCRA plot to assess the relative probability that an intervention was among the best options or superior to other interventions. The relative hazard ratio and mean difference between comparisons were displayed using a league table.

## Results

**Fig. 1:**
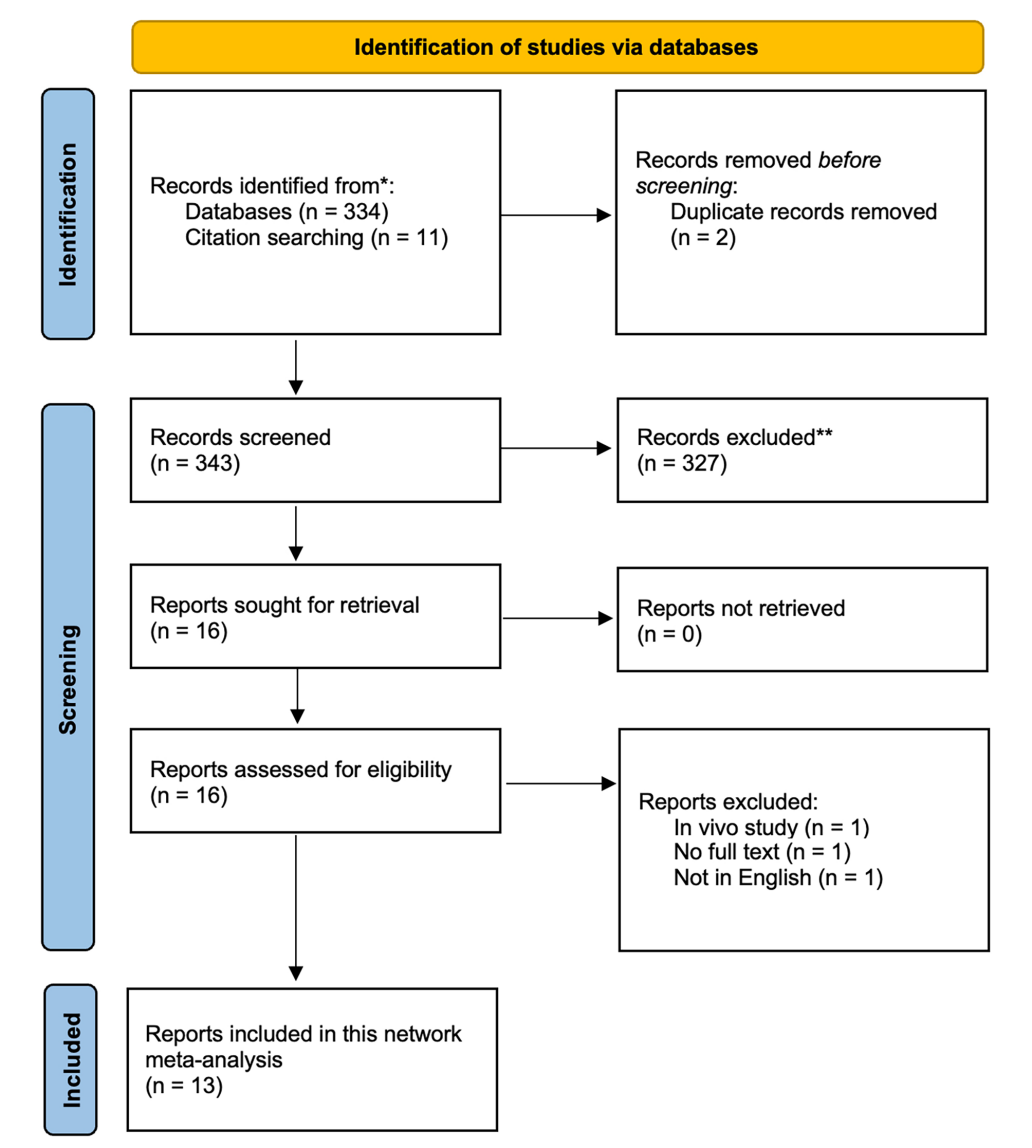
PRISMA flow chart. The literature search for this network meta-analysis was conducted according to the guidelines recommended by PRISMA

**Fig. 2:**
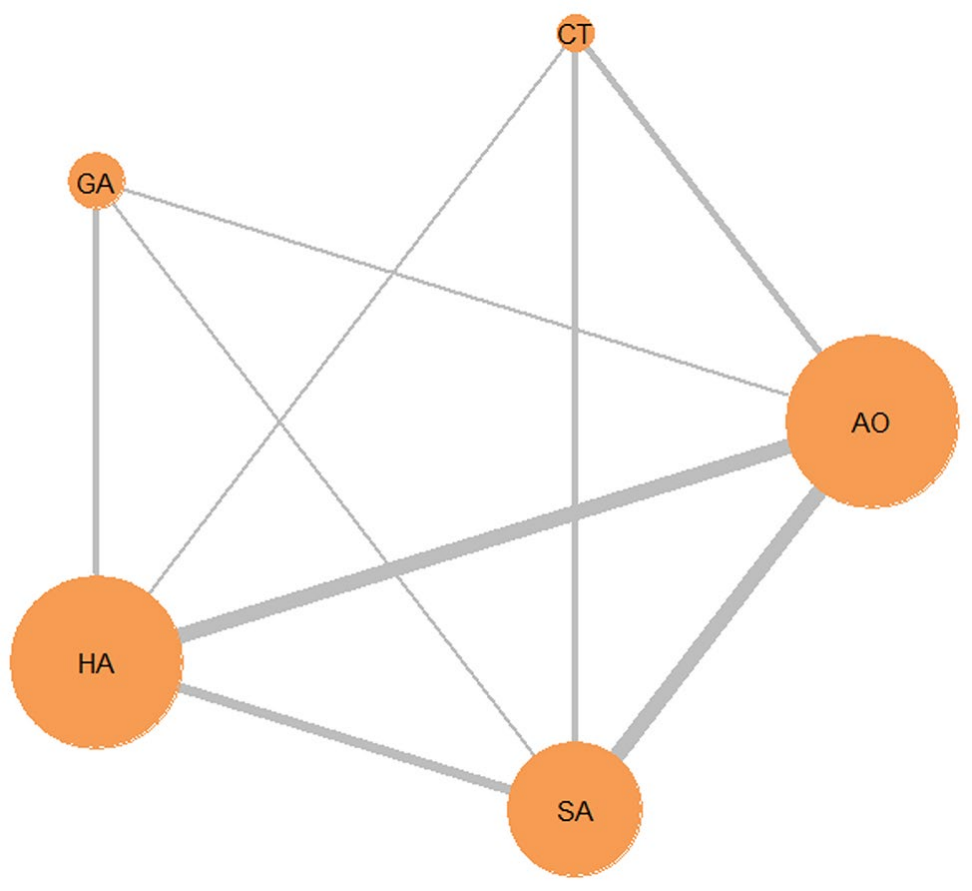
Network diagram illustrating the results of the trials comparing different conduits for liver transplantation. The size of each circle corresponds to the number of study participants. The number of trials comparing the respective pair of studies is directly proportional to the width of the lines (Abbreviations: CT, Celiac trunk; SA, Splenic artery; HA, Hepatic artery; AO, Aortic artery; GA, Branches of gastric artery)

Figure 1 depicts the literature search procedure. Once duplicate results were eliminated, 343 records were screened using titles and abstracts. Sixteen articles met the established inclusion criteria for full texts, although three of these were excluded with the reasons explained. The remaining thirteen articles were selected for further analysis. Table 1 presents the information and characteristics of the included studies. The mean follow-up time for this NMA was 51 months. Among the patients included, 72.8% were male and the mean age was 48.91 ± 6.12 years. The network of treatment comparisons of the available trials is shown in Fig. 2. The 13 studies included 5 interventions with 9521 patients in the network. Of these, 148 underwent anastomosis with the splenic artery, 1293 with the aortic artery, 19 with the celiac trunk, and 63 with branches of the gastric artery.

**Table 1:** Characteristics of study

Author	Year	Country	Study	Recipient Artery	Number of Patients	Age (Mean)	Sex (Male)	Follow-up (months)
Beaurepaire et al. [7]	2022	France	Retrospective	SA	26	58	16	70
				CT	12	56.5	9	32
				AO	91	52	59	62
				HA	1395	57	1079	61
Alim et al. [12]	2021	Turkey	Prospective	SA	16	49	NA	42
				GA	6	34	NA	42
				AO	649	51	NA	42
Llado et al. [13]	2019	Spain	Prospective	HA	1405	53	959	24
				SA	54	49	32	24
Rhu et al. [14]	2019	South Korea	Retrospective	HA	765	53	598	48
				GA	35	54	25	48
Kazemi et al. [15]	2017	Iran	Retrospective	SA	17	32	4	12
				AO	76	39	41	12
Dokmak et al. [16]	2015	France	Prospective	CT	7	48	2	15
				SA	2	48	2	24
				AO	2	50	2	24
Hibi et al. [4]	2013	USA	Retrospective	AO	267	54	165	89
				HA	1112	53	744	89
Cappadonna et al. [17]	2001	USA	Prospective	AO	37	47	21	32
				HA	168	49	90	38
Uchiyama et al. [18]	2010	Japan	Prospective	HA	313	51	152	12
				GA	22	51	7	12
Nikitin et al. [19]	2008	USA	Retrospective	AO	149	50	NA	240
				HA	2197	50	NA	240
Del Gaudio et al. [20]	2005	Italy	Retrospective	HA	313	51	152	12
				AO	22	51	7	12
Ullah et al. [21]	2003	Pakistan	Prospective	SA	512	48	67	42
				HA	101	46	355	39
Figueras et al. [22]	1997	Spain	Prospective	SA	23	NA	NA	18
				AO	12	NA	NA	18

*Abbreviations*: CT, Celiac trunk; SA, Splenic artery; HA, Hepatic artery; AO, Aortic artery; GA, Branches of gastric artery; NA, not available

**Fig. 3:**
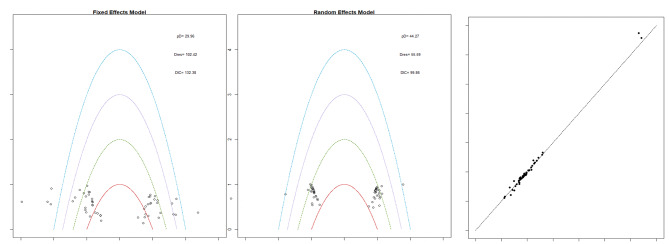
(**a**) fixed-effects model, (**b**) random-effects model, (**c**) consistency and inconsistency agreement

This study used a random analytical model. The DIC was substantially lower in the random effects model than in the fixed effects model. The fixed effects model revealed that the poor fit of the model mainly related to five points. Figure 3a and b show only one outlier in the random effects model. The consistency of the network was then evaluated by fitting the random effects inconsistency model and contrasting it with the consistency model. The data, except those of one or two locations, fall between the y and x lines, suggesting that the two models generally agree (Fig. 3c). Therefore, the consistency and random effects model were used for the meta-analysis.

### Intraoperative measures

**Fig. 4:**
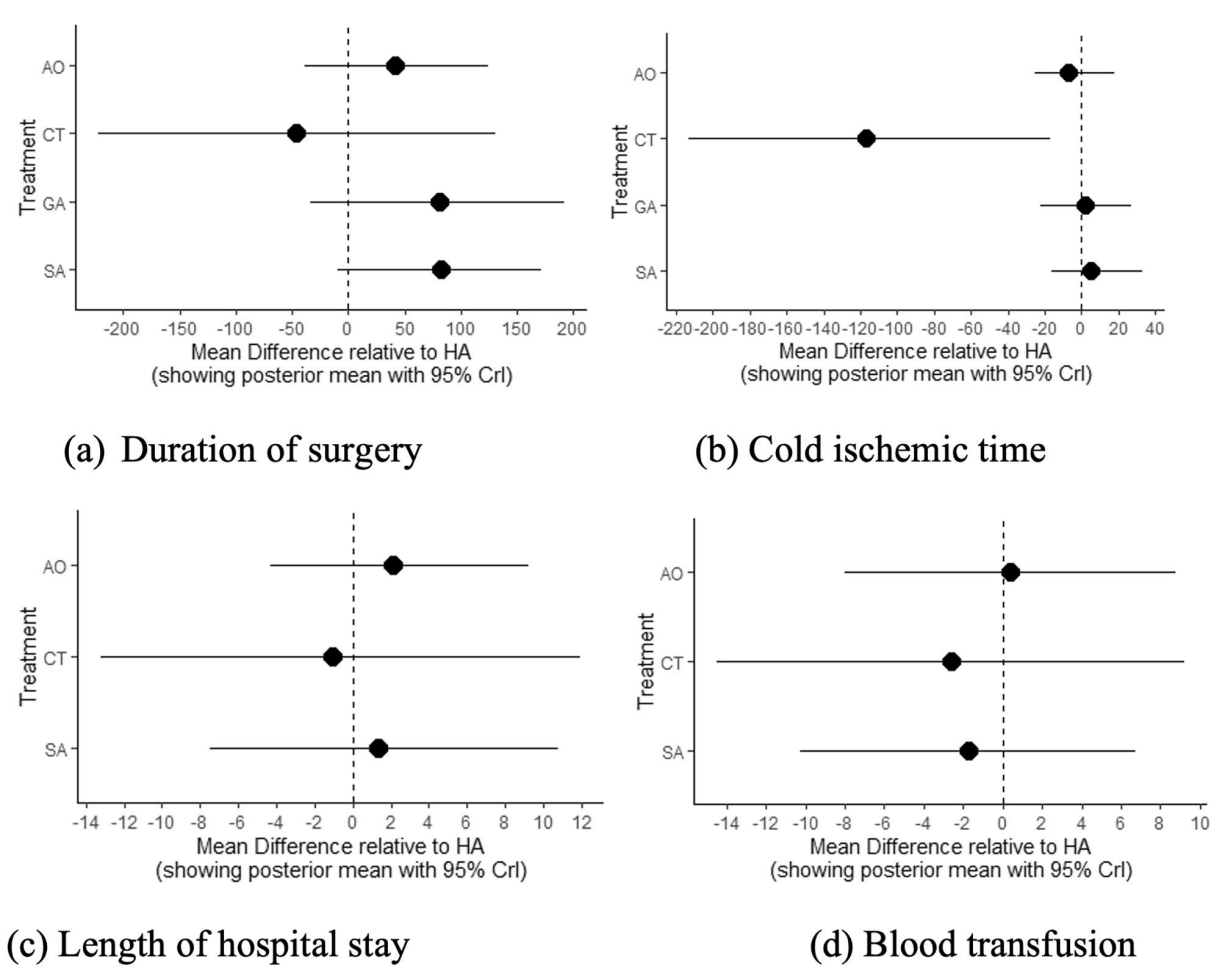
Forest plot of intraoperative measures among alternative conduits analyzed in this network meta-analysis. The bullets on the left indicate lower values than those on the right, and in terms of intraoperative measures, left-side positions are more advantageous. (**a**) Duration of surgery: celiac artery had the shortest duration; (**b**) Cold ischemic time: celiac artery had the shortest time; (**c**) Length of hospital stay: celiac artery had the shortest hospital stay; (**d**). Blood transfusion: celiac artery had the least need for blood transfusion. (Abbreviations: CT, Celiac trunk; SA, Splenic artery; HA, Hepatic artery; AO, Aortic artery; GA, Branches of gastric artery)

The shortest surgery duration for liver transplantation in liver recipients occurred when the celiac artery was used (MD -45.7, 95% CI -221 to 131). The time for this surgery was less than that of a standard hepatic artery anastomosis. In addition, these anastomosis locations (celiac MD -116, 95% CI -213 to -16.7), along with the aortic anastomosis (MD -7, 95% CI -25 to 17.6), showed the shortest cold ischemic periods compared to those of other locations. In contrast, anastomosis of the splenic artery revealed a longer operation duration and cold ischemic period compared to the other arteries used. Furthermore, the need for blood transfusions was minimal for celiac (MD -2.61, 95% CI -14.5 to 9.2) and splenic artery (MD -1.74, 95% CI -10.2 to 6.7) anastomoses compared to those required when the other arteries were used. The shortest hospital stay occurred in the group that underwent standard hepatic artery anastomosis, followed by celiac (MD -1.06; 95% CI -13.2 to 11.9) and splenic artery (MD 1.36; 95% CI -7.47 to 10.8) procedures (Fig. 4).

### Complications

**Fig. 5:**
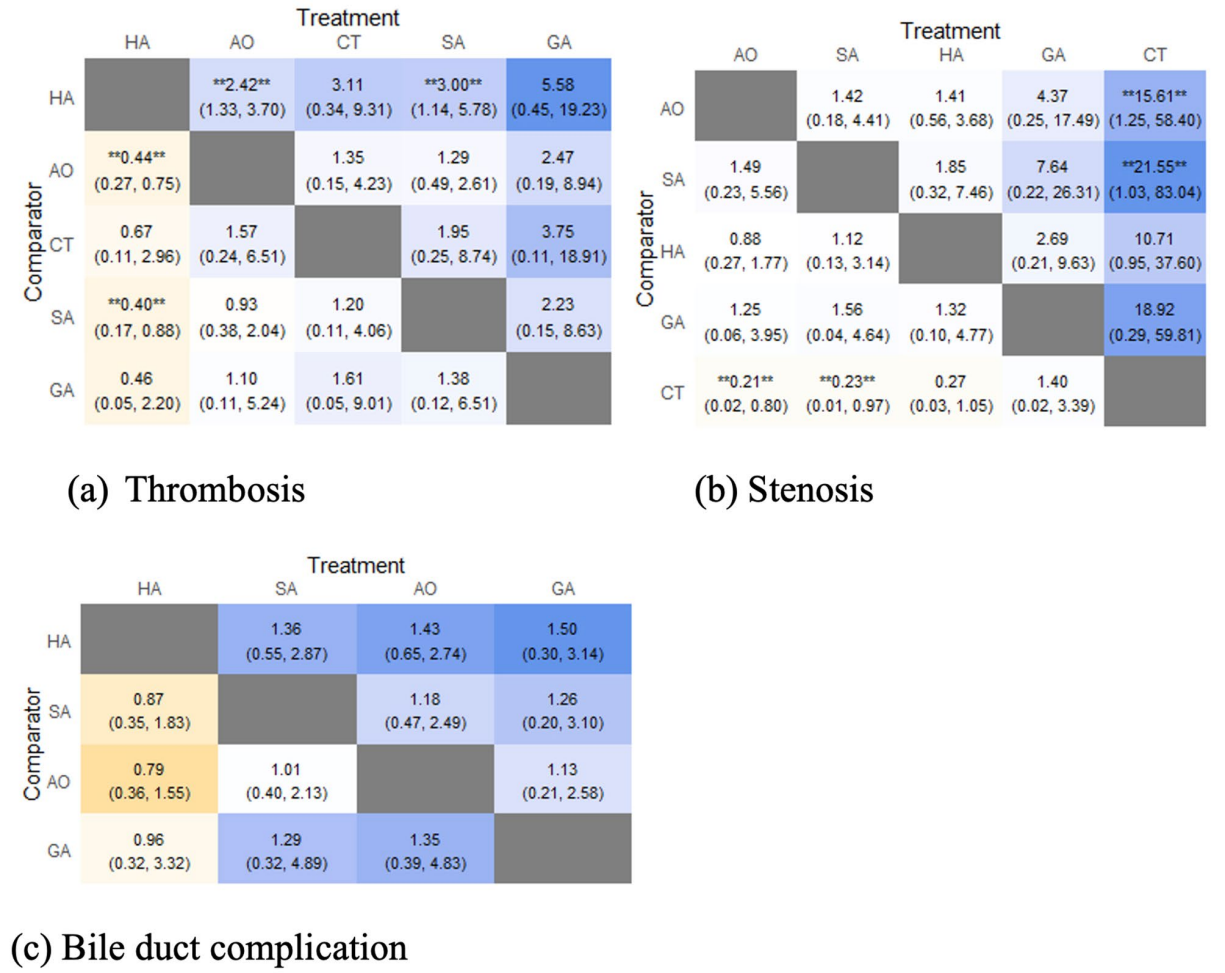
The complication rates of alternative conduits based on the findings of this network meta-analyses. Asterisks signify significance at *p* < 0.05. The values indicate the relative odds ratios between the variables (Abbreviations: SA, Splenic artery; HA, Hepatic artery; AO, Aortic artery; GA, Branches of gastric artery)

Of the arterial choices available, the standard anastomosis using the hepatic artery showed the lowest risk of thrombosis and bile duct complications. However, anastomosis of the aorta presented the least risk of stenosis (OR 0.88, 95% CI 0.27–1.77) followed by the splenic artery (OR 1.12, 95% CI 0.13–3.14). A low risk of thrombosis was demonstrated by anastomoses to the aortic (OR 0.44, 95% CI 0.27–0.75) and celiac (OR 0.4, 95% CI 0.17–0.88) vessels. Bile duct complications were minimal when anastomosis was performed using the hepatic or splenic arteries (OR 0.79, 95% CI 0.36–1.55) (Fig. 5).

### Survival

The donor hepatic artery showed superior graft survival compared to the other anastomoses. Relatively good survival occurred with the use of branches of the gastric vessels (OR 1.03; 95% CI 0.96–1.23) and splenic arteries (OR 1.08; 95% CI 0.96–1.23).

**Fig. 6:**
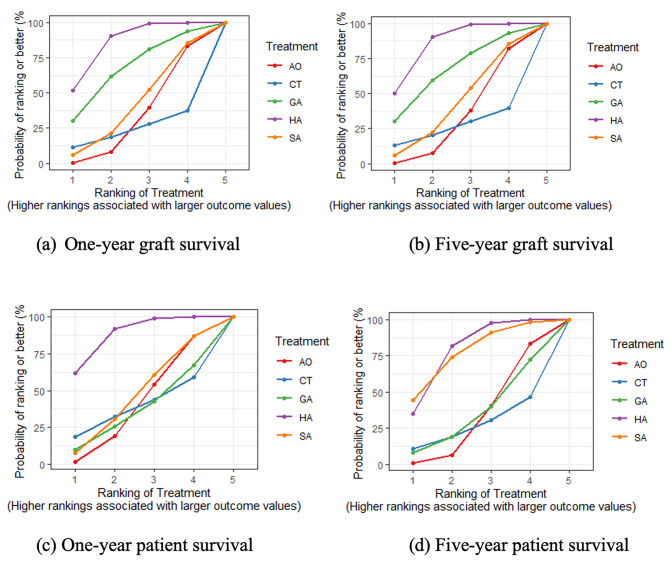
Surface under the cumulative ranking curve (SUCRA) plot of patient and graft survival. The top line of the graph shows the highest ranking, which indicates the treatment with the best survival relative to the others (Abbreviations: AO, Aortic artery; CT, Celiac trunk; GA, Branches of gastric artery; SA, Splenic artery; HA, Hepatic artery)

Standard anastomosis also produced the best patient survival results compared to those with the use of other arteries. The longest patient survival duration occurred with the alternative anastomosis using the splenic (1-year survival OR 1.09, 95% CI 0.94–1.26; 5-year survival OR 1, 95% CI 0.83–1.22) and aortic (1-year survival OR 1.10, 95% CI 0.99–1.25; 5-year survival OR 1.13, 95% CI 0.85–2.15) arteries (Fig. 6).

## Discussion

The standard recipient hepatic artery was used in 71.4–88% of the patients in the chosen studies [23, 24]. If the native hepatic artery of the recipient is not viable, surgeons must find alternate innovative strategies to maintain sufficient blood flow to the transplanted liver. Each approach presents unique advantages and challenges and requires a meticulous assessment of the condition of the patient and the surgical expertise available to determine the most suitable option.

Most studies used the recipient aorta, either the abdominal aorta or common iliac artery, as the first alternative due to its location and large diameter [25]. However, recent studies reported high rates of thrombosis after surgery and impaired graft survival [17, 20]. Furthermore, this procedure required additional dissection, clamping of the aorta, and a longer trajectory than the standard method [26]. Therefore, surgeons attempted to use the splenic artery as an alternative since this artery is readily accessible during liver transplantation surgery and can be easily identified and anastomosed to the vasculature of the recipient. This procedure was technically feasible and added a level of flexibility for the transplant surgeon [18]. Anastomosis to branches of the gastric artery of the recipient was also performed because, although the diameter is small, living donor liver transplantations allow the surgeons the ability to choose the branches that fit the diameter of the donor hepatic artery [16]. The early patency of these shunts is good; however, the long-term results are unclear [18]. Direct anastomosis to the celiac trunk was rarely performed due to the technical challenges and potential risks associated with this method [27].

The shortest operating time for liver transplantation occurred when the celiac artery was used. The standard hepatic artery and celiac artery anastomoses showed the shortest cold ischemic times compared to other locations, along with the aortic anastomosis (MD -7, 95% CI -25 to 17.6). Anastomosis of the splenic artery required a longer duration of operation and cold ischemic time compared to the other anastomosis locations. However, the need for blood transfusions for celiac (MD -2.61, 95% CI -14.5 to 9.2) and splenic artery (MD -1.74, 95% CI -10.2 to 6.7) anastomoses was minimal when compared to those of other anastomoses. The shortest hospital stay occurred in the standard hepatic artery anastomosis group, followed by the celiac (MD -1.06; 95% CI -13.2 to 11.9) and splenic artery (MD 1.36, 95% CI -7.47 to 10.8) groups.

The complications encountered with the aortic anastomosis included thrombosis (OR 0.44, 95% CI 0.27–0.75), stenosis (OR 0.88, 95% CI 0.27–1.77), and overall biliary tract obstruction (OR 1.43; 95% CI 0.65–0.74). The splenic artery showed a low risk of stenosis (OR 1.12, 95% CI 0.13–3.14) and complications in the biliary tract (OR 0.79, 95% CI 0.36–1.55); however, the risk of anastomotic thrombosis in the splenic artery was relatively high (OR 3, 95% CI 1.14–5.78).

**Fig. 7:**
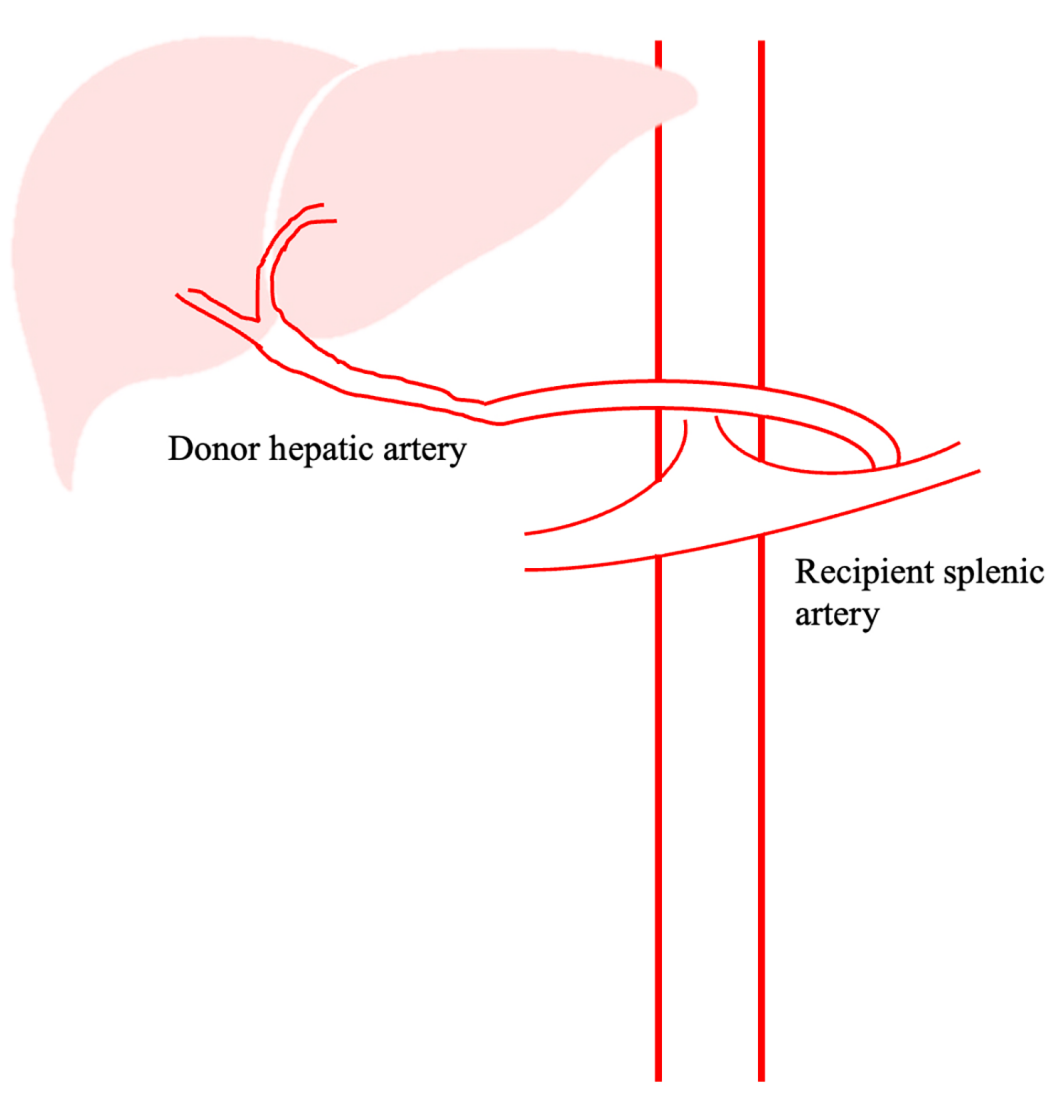
Anastomosis between donor hepatic artery and recipient splenic artery in liver transplantation

Graft survival with aortic anastomosis was inferior to anastomoses using other vessels, with the celiac artery revealing the poorest graft survival rates. This is consistent with the lower patient survival observed with this method compared to other blood vessel anastomoses. The survival analysis indicated that the use of gastric artery branches produced the best graft survival. The left gastric artery was mobilized from the lesser curve proximally to the celiac artery and superficialized with a natural rightward curve for tension-free anastomosis, which improved survival. Although the size was small, the technique of spatulation allowed end-to-end anastomosis between the left gastric artery and the donor hepatic artery [28]. Despite these results, patient survival was the second poorest using this artery compared to the others included in this NMA. This may relate to the complications of thrombosis, stenosis, and high biliary tract observed with this method. In contrast, the splenic artery showed relatively good graft patient survival rates (Fig. 7).

These results indicate that surgeons must carefully evaluate each case and select the most appropriate technique to ensure successful liver transplantation and optimal graft function. Continued advancements in surgical techniques and further research in alternative conduits can improve outcomes for liver transplantation patients with unusable recipient hepatic arteries.

## Conclusion

The recipient splenic artery can be considered an alternative anastomosis site for liver transplantations if the hepatic artery is not viable.

## Data Availability

All data generated or analyzed during this study are included in this published article.
